# Effects of TGF-beta signalling inhibition with galunisertib (LY2157299) in hepatocellular carcinoma models and in *ex vivo* whole tumor tissue samples from patients

**DOI:** 10.18632/oncotarget.4308

**Published:** 2015-05-27

**Authors:** Maria Serova, Annemilaï Tijeras-Raballand, Célia Dos Santos, Miguel Albuquerque, Valerie Paradis, Cindy Neuzillet, Karim A. Benhadji, Eric Raymond, Sandrine Faivre, Armand de Gramont

**Affiliations:** ^1^ AAREC Filia Research, Boulogne-Billancourt, France; ^2^ INSERM U728, Department of Medical Oncology, Beaujon University Hospital (AP-HP - PRES Paris 7 Diderot), Clichy, France; ^3^ INSERM U773, Department of Pathology, Beaujon University Hospital (AP-HP - PRES Paris 7 Diderot), Clichy, France; ^4^ Eli Lilly and Co, Neuilly-sur-Seine, France

**Keywords:** TGF-β inhibition, hepatocellular carcinoma, galunisertib, sorafenib, SMAD

## Abstract

Galunisertib (LY2157299) is a selective ATP-mimetic inhibitor of TGF-β receptor (TβR)-I activation currently under clinical investigation in hepatocellular carcinoma (HCC) patients. Our study explored the effects of galunisertib *in vitro* in HCC cell lines and *ex vivo* on patient samples. Galunisertib was evaluated in HepG2, Hep3B, Huh7, JHH6 and SK-HEP1 cells as well as in SK-HEP1-derived cells tolerant to sorafenib (SK-Sora) and sunitinib (SK-Suni). Exogenous stimulation of all HCC cell lines with TGF-β yielded downstream activation of p-Smad2 and p-Smad3 that was potently inhibited with galunisertib treatment at micromolar concentrations. Despite limited antiproliferative effects, galunisertib yielded potent anti-invasive properties. Tumor slices from 13 patients with HCC surgically resected were exposed *ex vivo* to 1 μM and 10 μM galunisertib, 5 μM sorafenib or a combination of both drugs for 48 hours. Galunisertib but not sorafenib decreased p-Smad2/3 downstream TGF-β signaling. Immunohistochemistry analysis of galunisertib and sorafenib-exposed samples showed a significant decrease of the proliferative marker Ki67 and increase of the apoptotic marker caspase-3. In combination, galunisertib potentiated the effect of sorafenib efficiently by inhibiting proliferation and increasing apoptosis. Our data suggest that galunisertib may be active in patients with HCC and could potentiate the effects of sorafenib.

## INTRODUCTION

Hepatocellular carcinoma (HCC) is one of the most deadly cancers whose incidence follows a rising trend worldwide [1]. It is also a disease with very few therapeutic options at advanced stage, in which sorafenib remains the only approved drug since 2007 based on the results of two randomized studies [2, 3]. Most HCCs develop on pathological livers due to either hepatitis B (HBV) or C (HCV) infection, alcohol consumption (cirrhosis), inherited metabolic disease and dysmetabolic syndrome such as nonalcoholic fatty liver disease. In recent years, TGF-β pathway has gained considerable interest due to its pleiotropic role in regulating cell growth, differentiation, apoptosis, and motility at the tumor level, as well as extracellular matrix production, angiogenesis, and cellular immune response at the tumor microenvironment level [4-6]. TGF-β signals through binding to TGF-β type II receptors (TGFBRII) that dimerize with TGF-β type I receptors (TGFBRI) and activates the TGF-β dependent canonical signal transducers SMADs. Phosphorylated Smad2 and Smad3 then form a trimeric complex with Smad4 that translocates to the nucleus interacting with various transcription factors to activate or repress hundreds of target genes. At Smad levels, TGF-β pathway integrates signaling from integrins, Notch, Wnt, TNF-α, or EGF-dependent pathways as well as signals from cellular processes such as the cell-cycle or apoptosis machineries.

TGF-β exerts a dual role on HCC tumorigenesis displaying tumor suppressive properties at early stage while promoting tumor progression at later stage [6, 7]. The switch from cytostatic to pro-tumorogenic properties is thought to depend on TGF-β pathway alterations as well as signal integration from other pathways. Despite some occurrences affecting the TGFBRII gene, mutations in the TGF-β pathway are rare in HCC [8, 9]. In few HCC subsets, low expression levels of TGF-β receptors or of the inhibitory protein Smad7, as well as increased expression of Smad4, have been correlated with poor prognosis, but data need to be consolidated to reach a definitive consensus [10-14]. Therefore, crosstalks and TGF-β coupling with other signalling pathways is thought to be critically important for tumor progression, notably to circumvent the initial cytostatic effect of the TGF-β pathway. Early events of HCC carcinogenesis may thus require lowering TGF-β activity while relieving growth inhibition. As such, increased expression of Smad7 and repression of Smad2 and Smad3 expression are characteristics of the early TGF-β signature as defined by Coulouarn and colleagues [15] whereas mutations in the cell-cycle inhibitor p16^INK4^ are present in most HCC [16]. In contrast, late carcinogenesis events, such as cancer dissemination, are characterized by cytoskeleton reorganization, modification of cell-cell adhesion, matrix remodelling and migration, which imply modulation of several genes such as Vimentin and β1-integrin whose expressions have been described in the late TGF-β signature [17]. TGF-β pathway activation has been shown to correlate with epithelial-to-mesenchymal transition (EMT) which itself is associated with poor prognosis [18]. Increased expression of circulating TGF-β1 and alpha feto-protein (AFP) expression are considered hallmarks of the disease [19, 20]. TGF-β1 inhibition reduces cell invasiveness, possibly via partial EMT reversion *in vitro*, and reduction of both TGF-β1, and AFP plasma levels have been shown to be associated with effective therapy in HCC patients [17, 21]. Accumulating evidence shows that TGF-β can activate and cooperate with pathways involved in invasion and metastasis (e.g., MAPK, PI3K/AKT/mTOR, NF-κB, Notch, Wnt, and CXCR4) through Smad2/3-dependent and -independent signalling mechanisms [22].

Galunisertib (LY2157299), a selective ATP-mimetic inhibitor of TGFBRI, is the only TGF-β pathway inhibitor currently under clinical investigation in HCC patients (NCT01246986). In a recent paper, galunisertib has been shown to efficiently inhibit the expression of p-Smad2 as well as invasion but not proliferation in 3 HCC models *in vitro* [23, 24]]. The aim of this study was to characterize galunisertib effects on a different set of HCC models for proliferation and invasion and investigate its effect on canonical and noncanonical TGF-β signaling. We also tested the potential combinability of galunisertib with sorafenib in cells and *ex vivo* models, i.e., in fresh tumor explants maintained alive for several days. Using HCC fresh tumor explants to test TGF-β inhibitors has not been described yet and may represent an interesting way to test potential new therapeutics in HCC.

## RESULTS

### Characterization of HCC models for TGF-β dependency

**Figure 1:**
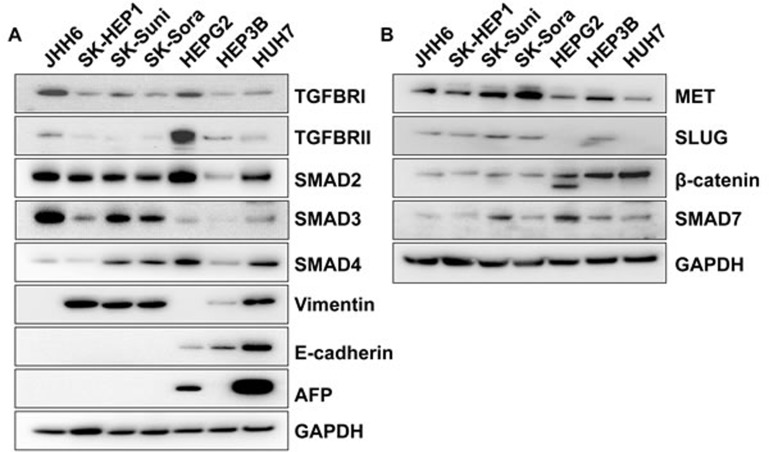

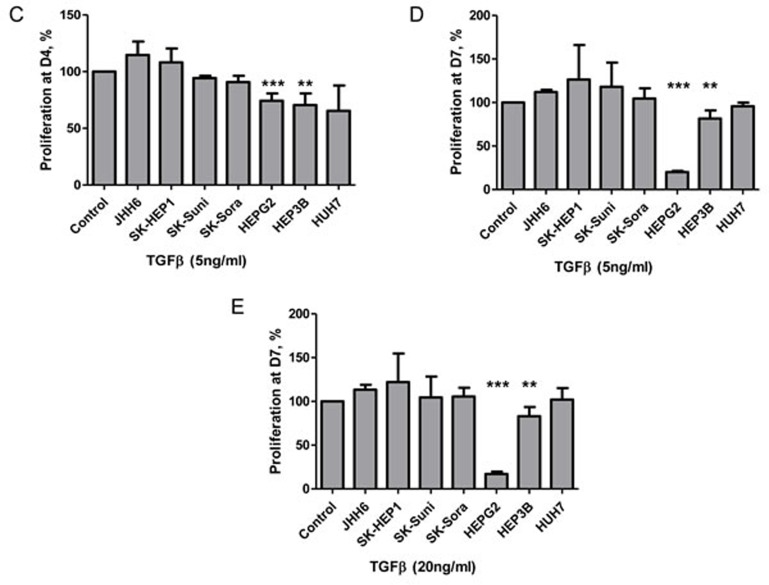
Characterization of HCC cell lines **A.** Protein levels of TGFBR1, TGFBR2, SMAD2, SMAD3, SMAD4, AFP, E-cadherin and Vimentin was detected by Western blot in a panel of cell lines; **B.** Characterization of hepatocarcinoma cell lines for protein expression of c-MET, Slug, β-catenin, and SMAD7 by Western blot; **C.** Antiproliferative effects of 5 ng/mL TGF-β after 4 days of exposure on the panel of HCC cell lines; **D.** Antiproliferative effects of 5 ng/mL TGF-β after 7 days of exposure on the panel of HCC cell lines; **E**. Antiproliferative effects of 20 ng/mL TGF-β after 7 days of exposure on the panel of HCC cell lines.

Given the dual role of TGF-β, displaying either cytostatic or pro-tumorogenic properties, we first characterized our 7 HCC cell lines for TGF-β pathway protein expression (TGFΒR1, TGFΒR2, Smad2, Smad3, Smad4, Smad7) and TGF-β dependent effects on cell proliferation in order to select the most appropriate models to study TGF-β inhibitors. We also characterized the cell panel for expression of mesenchymal (Vimentin, c-MET, and Slug) or epithelial (E-cadherin and β-catenin) markers and for AFP expression using Western blot (Figure 1A and 1B).

JHH6 had the particularity to express the highest levels of TGFBR1, Smad2, and Smad3 without expressing Vimentin or E-cadherin but expressing c-MET and Slug. In contrast, SK-HEP1 was characterized by low expression of most TGF-β pathway-related proteins with the exception of Smad2. SK-HEP1 displayed a strong mesenchymal phenotype with expression of Vimentin, c-MET, and Slug, without expression of E-cadherin or β-catenin. Drug-tolerant cell lines, SK-Suni and SK-Sora, displayed a protein expression profile similar to the parental SK-HEP1 but increased Smad3 and Smad4 expression as well as an exacerbated mesenchymal phenotype characterized by high c-MET expression; of note, SK-Suni displayed increased expression of the inhibitory Smad7 compared to SK-Sora (Figure 1A and 1B). All these cell lines were negative for AFP expression. The other cell lines, HepG2, HuH7 and Hep3B, displayed an epithelial phenotype, i.e., expression of E-cadherin and β-catenin and low or no expression of c-MET and Slug. HepG2 was specifically characterized by its expression of both TGFBR1 and TGFBR2, as well as Smad7. In contrast, HuH7 and Hep3B expressed very low levels of TGF-β receptors. Both HepG2 and HuH7 expressed Smad2 and Smad4 but a low level of Smad3 whereas Hep3B was characterized by low expression of all Smads (Figure 1A and 1B). Among all these models, HepG2 and HuH7 were the only cell lines to express AFP (Figure 1A and Supplementary Figure 1).

Expression of the TGF-β ligands TGF-β1, TGF-β2, and TGF-β3 was assessed by qRT-PCR. TGF-β1 and TGF-β2 expression levels were increased in SK-HEP1, SK-Suni, and SK-Sora compared to HepG2 and Hep3B. TGF-β3 expression displayed a reverse pattern with higher expression in HepG2 and Hep3B than in SK-Hep1 cell lines (data not shown).

Differential expression pattern of E-cadherin, Vimentin, c-MET, Slug, and TGF-β1 suggested that SK-HEP1, SK-Suni, SK-Sora, and JHH6 belonged to the late TGF-β signature subgroup whereas HepG2, HuH7 and Hep3B belonged to the early TGF-β signature subgroup of HCC models as previously described (15). Since early TGF-β signature HCC models are expected to be sensitive to TGF-β antiproliferative effects but not late TGF-β signature models, we tested TGF-β growth inhibition on our 7 cell lines (Figure 1C-1E). SK-HEP1, SK-Suni, SK-Sora, and JHH6 did not display TGF-β dependent cytostasis. JHH6, which has the particularity of expressing neither E-cadherin nor Vimentin and was very weakly responsive to TGF-β pathway activation might be more representative of the negative TGF-β rather than the late signature subgroup. Overall, these results support the use of late TGF-β signature cell lines as the most appropriate models to study the direct effects of TGF-β inhibitors (Table 1).

**Table 1:** Cell lines characterization

Cell lines	Phenotype	TGF-β signature	TGF-β growth inhibition	AFP	HBV
**SK-HEP1**	Mesenchymal, poorly differentiated		No	-	-
**SK-Suni**	Mesenchymal	Late	No	-	-
**SK-Sora**	Highly mesenchymal		No	-	-
**JHH6**	Mesenchymal, undifferentiated	Late	No	-	-
**HepG2**	Epithelial, partially differentiated	Early	Yes	+	-
**Hep3B**	Epithelial, well differentiated		Yes	-	+
**HuH7**	Epithelial, well differentiated		Yes	+	-

### Galunisertib effects on canonical and noncanonical TGF-β signalling

To study the effects of galunisertib on intracellular signalling, HCC cells were incubated in serum-free medium overnight and treated with 5 ng/mL of TGF-β for 1 hour followed by the addition of 1 or 10 μM galunisertib for 5 and 24 hours. Concentrations were selected to be in the range of observed plasma exposure [25].

**Figure 2:**
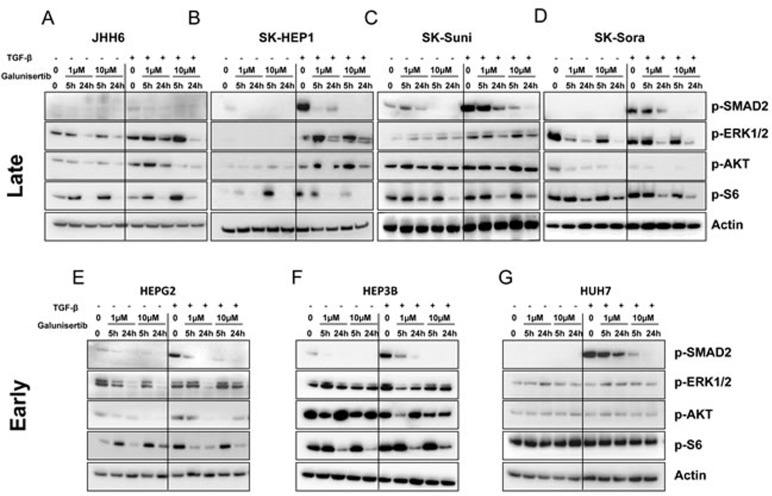
Effects of galunisertib on canonical and noncanonical TGF-β signalling Protein expression was evaluated by Western blot after cell treatment with 1 and 10 μM galunisertib with or without 5 ng/mL TGF-β; JHH6 **A.** SK-HEP1 **B.** SK-Suni **C.** SK-Sora **D.** HepG2 **E.** Hep3B **F.** and HuH7 **G.** cells.

Addition of TGF-β induced substantial activation of Smad-dependent canonical pathways as shown by the increased expression of p-Smad2 in all models except in JHH6 cell line (Figure 2). Moreover, TGF-β addition induced slight activation of the noncanonical PI3K/AKT and/or MAPK pathways in SK-HEP1, SK-Suni, HepG2, and JHH6 but not in the other cell lines. Addition of galunisertib reduced the expression levels of p-Smad2 in all cell lines in a dose and time-dependent manner independently of TGF-β induction. In SK-HEP1, HepG2, and Hep3B, a significant reduction of p-Smad2 expression was observed at early time point (5 hours) for 1 μM galunisertib. After 24 hours exposure of 10 μM galunisertib, only residual p-Smad2 expression was detectable. In contrast to p-Smad2 expression, galunisertib moderately affected PI3K/AKT and MAPK pathways. Some transient effects were observed at 5 hours, but after 24 hours exposure to galunisertib, expression of p-ERK1/2 and p-AKT were unchanged compared to baseline levels in SK-HEP1, SK-Suni, Hep3B, and HuH7 (Figure 2). In SK-Sora, HepG2, and JHH6, 24-hour exposure of galunisertib significantly inhibited p-ERK1/2 and p-AKT expression (Figure 2C and 2D). Interestingly, PI3K/AKT pathway inhibition by galunisertib was independent from exogenous TGF-β stimulation. Moreover, after 24 hours of galunisertib exposure, potent inhibition of p-S6, a downstream target of mTOR, was observed in all cell lines except HUH7. In conclusion, galunisertib displayed potent inhibition of canonical TGF-β signalling in 6 out of 7 HCC models and selected inhibition of noncanonical pathways in several models. The exquisite sensitivity of canonical and noncanonical TGF-β pathways signalling inhibition by galunisertib in the sorafenib-resistant cell line SK-Sora warranted exploration of galunisertib in combination with sorafenib (see below).

### Galunisertib exerted limited antiproliferative but strong anti-invasive effects

**Figure 3:**
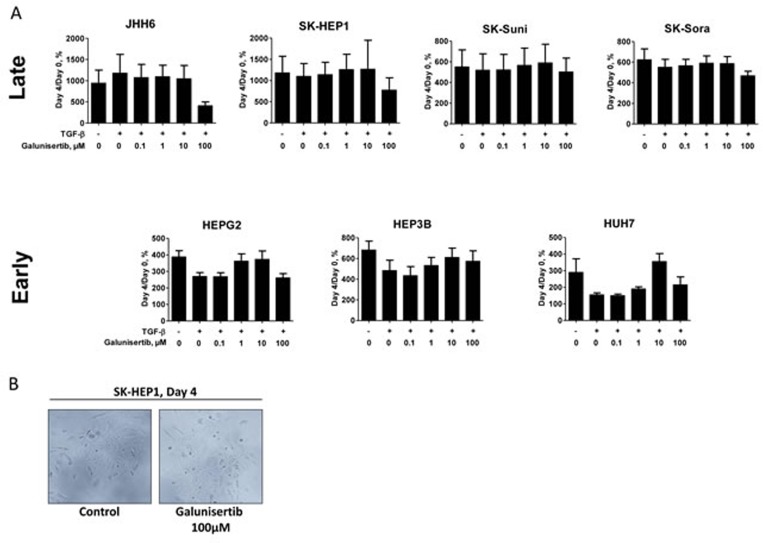

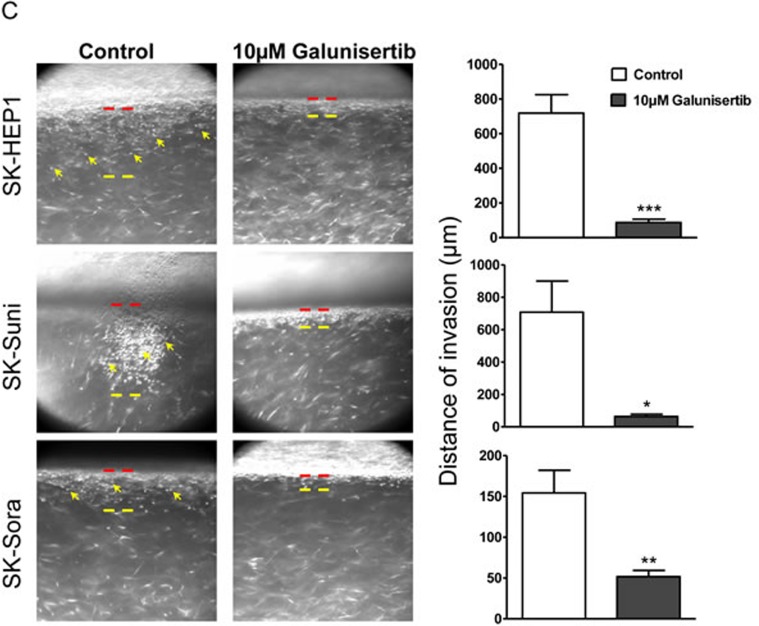
Antiproliferative and anti-invasive effects of galunisertib **A.** Antiproliferative effects of galunisertib with and without TGF-β (5 ng/mL) on a panel of HCC cell lines; **B.** Effects of 4 days exposure to 100μM galunisertib, on SK-HEP1 morphology; **C.** Epifluorescent microscopy of the *in vitro* OptiCell invasion assay on HCC SK-HEP1, SK-Suni, and SK-Sora cells under different culture conditions. Control: no treatment (left panel); Treatment with 10 μM exogenous galunisertib (right panel). Arrows indicate cancer cells.

Cancer cell lines were exposed continuously to 0.1, 1.0, 10, and 100 μM galunisertib in presence or absence of TGF-β (5 ng/mL) and assessed for inhibition of cellular proliferation after 72- and 96-hour incubations using the MTT assay. In the late TGF-β signature cell group, no effects on proliferation were observed in SK-HEP1, SK-Suni, SK-Sora, and JHH6 exposed to concentrations of galunisertib below 100 μM with or without TGF-β (Figure 3A, Supplementary Figure 2). In the early TGF-β signature cell group, HepG2, Hep3B, and HuH7 cell lines were sensitive to TGF-β dependent growth inhibition and also displayed limited sensitivity to galunisertib (Figure 3A). In these cells, 0.1 to 10 μM galunisertib seemed to reverse TGF-β cytostatic effects in a dose-dependent manner. High galunisertib concentrations (100 μM) displayed TGF-β independent antiproliferative effects in most cells without exhibiting cytotoxic effects (Figure 3A and Supplementary Figure 2). Given limited antiproliferative effects *in vitro*, we decided to examine the effects of galunisertib on extracellular matrix (ECM)-degrading capabilities of the invasive models of our HCC panel, i.e., SK-HEP1, SK-Suni, and SK-Sora and their ability to penetrate the basement membrane and migrate into its supporting connective tissue. We used the OptiCell assay to directly visualize the invasion process (see Material and Methods). Fibroblasts embedded into type I collagen secreted a high level of TGF-β (1 ng/mL after 3 days) as detected by ELISA assay (data not shown). Direct monitoring of the multilayer cell construct showed that SK-HEP1, SK-Suni, and SK-Sora cells were able to penetrate into the collagen/fibroblasts gel after 7 days (Figure 3C). Pretreatment with 10 μM galunisertib strongly decreased cells migration into the gel. These results confirmed that galunisertib displays strong anti-invasive properties on HCC cell lines.

### *In vitro* combinations of galunisertib with sorafenib

**Figure 4:**
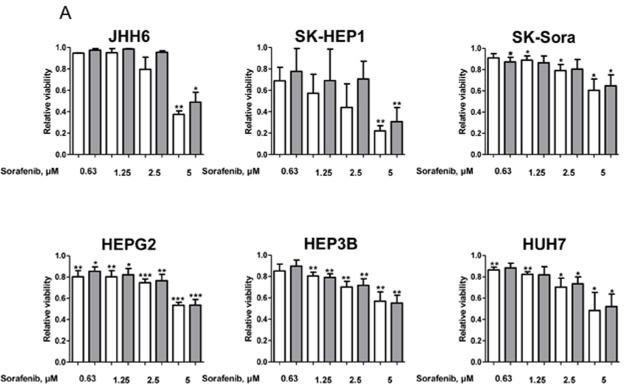

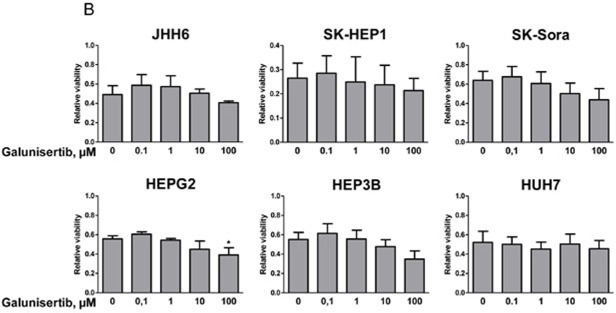
Cytotoxic effects of the galunisertib/sorafenib combination **A.** Relative cell viability after 72 hours exposure to 0.63, 1.25, 2.5, and 5 μM sorafenib with (gray bars) and without (white bars) 5 ng/mL TGF-β of JHH6, SK-HEP1, SK-Sora, HepG2, Hep3B, and HuH7 cells. **B.** Effects of 5 μM sorafenib combined with 0.1, 1, 10, and 10 μM galunisertib in the presence of 5 ng/mL TGF-β. Data represent the means +/− SD of 3 independent experiments.

We further explored the effects of galunisertib in combination with sorafenib in our models. We first determined the sorafenib sensitivity profile of our cell-line panel in presence or absence of TGF-β (Figure 4A). All cell lines displayed a dose-dependent sensitivity towards increasing sorafenib concentrations that was not influenced by exogenous TGF-β addition. SK-HEP1 appeared to be the most sensitive cell line, SK-Sora and JHH6 being the most resistant cells of our panel. HepG2, Hep3B, and HuH7 displayed similar intermediate sensitivity. To explore the potential of combining galunisertib with sorafenib in HCC cells, cell lines were simultaneously exposed to 0.1, 1, 10, and 100 μM galunisertib with 5 μM sorafenib in presence of TGF-β for 72 hours (Figure 4B). Galunisertib displayed a slight dose-dependent potentiation of sorafenib in SK-Sora, HepG2, and Hep3B cell lines but not in JHH6, SK-HEP1, and HuH7 cell lines.

### *Ex vivo* effects of galunisertib on tumor explants from hepatocellular carcinoma patients

**Figure 5:**
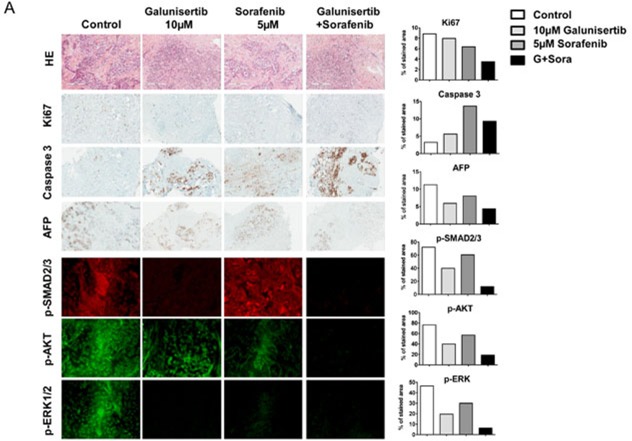

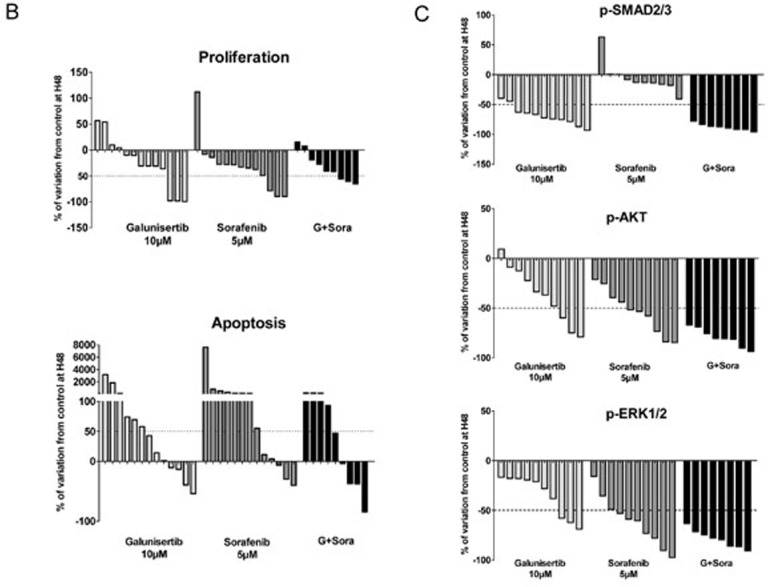
Effect of galunisertib on whole tumor tissue samples from patients with HCC Tumor slices were incubated 48 hours with 10 μM galunisertib and/or 5 μM sorafenib and then stained with HE, Ki67, caspase-3, AFP, p-SMAD2/3, p-AKT, and p-ERK antibodies. **A.** IHC pictures of the mentioned protein staining; graphs display protein expression quantification for the slide; **B.** Waterfall plot of the IHC analysis of samples from 13 HCC patients treated *ex vivo* with galunisertib, sorafenib, or a combination of both treatments; **C.** Waterfall plot of the IF analysis of samples from 13 HCC patients treated *ex vivo* with galunisertib, sorafenib, or a combination of both treatments.

Since the importance of the TGF-β pathway heavily relies on its tumour microenvironment, we used an *ex vivo* methodology in which explanted tumor slices are maintained alive in culture and treated with pharmacologically active drug concentrations. Different experimental conditions are intended to be reproduced between each tumor sample with the limitation of the number of slices available (Figure 5). After patients’ consents, fresh tumor samples were obtained from the surgery department. A total of 13 patient samples were analyzed. Clinical and pathological patients’ characteristics are summarized in Table 2. Tumors were sliced and incubated 48 hours with 10 μM galunisertib with or without sorafenib. After incubation, the slices were embedded in paraffin, HE stained, and analyzed by IHC for proliferation (MIB1/Ki67), apoptosis (caspase-3), AFP expression, and by immunofluorescence for downstream canonical and noncanonical TGF-β signalling, i.e., p-SMAD2/3, p-ERK1/2, and p-AKT expression. Figure 5A represents typical results obtained with HCC tumors. Significant decrease of MIB1/Ki67 staining was observed in patient samples treated with galunisertib (54%, 7 out of 13 samples displayed >25% decrease in protein expression), sorafenib (77%, 10/13) or both (67%, 6/9) (Figure 5B and Supplementary Table 1). In addition, a significant increase of caspase-3 staining was observed after galunisertib treatment (54%, 7 out of 13 samples displayed >25% increase in protein expression), sorafenib (62%, 8/13) or both (56%, 5/9).

**Table 2:** Patients’ characteristics

Patient ID	Sexe	Age	Etiology	Tumor size(cm)	Satellite nodules	Vascular invasion	Tumor differentiation	AFP levels	
								Serum (>20 ng/ml)	Tumor
01	M	39	HBV	2.8 × 2.5	No	Yes	Mixte	Normal	No
02	M	74	NASH	3.8 × 4	No	Yes	Moderately	N	N
03	M	44	HBV	20	Yes	Yes	Moderately	High	High
04	M	37	HBV	6 × 4,5 × 4	Yes	Yes	Moderately	N	N
05	M	54	HBV, HIV	19 × 17 × 7,5	Yes	Yes	Well /moderately	High	N
06	M	38	HBV	4 × 5 × 2	Yes	Yes	Moderately	ND	N
07	F	48	ND	18 × 18 × 6	Yes	Yes	Well /moderately	ND	N
08	M	63	Hemocromatosis	4.5 × 4.5 × 3.5	No	No	Well	N	N
09	M	68	HBV	3	No	Yes	Moderately	High	High
10	M	76	HCV	6	Yes	Yes	Moderately	High	N
11	M	63	HBV	10 × 7 × 6	Yes	Yes	Moderately	N	N
12	F	58	ND	16 × 12 × 17	Yes	Yes	Poorly	High	N
13	M	48	HBV	16	Yes	Yes	Moderately	High	N

To determine whether responsive tumors may belong to the late TGF-beta signature subgroup we assessed the expression of c-MET at baseline, Vimentin being detected at similar levels across all control tumor samples. c-MET expression varied from 17.8% positivity to 71.1% (median = 6.9%) and High c-MET expression was defined as positive expression ≥46.9%. Response parameters (proliferation, apoptosis, pSMAD2/3, pAKT pERK) were not different between the high and low c-MET groups despite a trend between an association of high c-MET expression with increased apoptosis induction in the Galunisertib+sorafenib subgroup (*p* = 0.08). However, the small number of tumor explants in this study inherently limits the significance of the results, which must be confirmed in larger studies.

In 2 IHC AFP-positive patient samples (patients 03 and 09), AFP staining slightly decreased after 48 hours of treatment with galunisertib and galunisertib/sorafenib combination but not with sorafenib alone (Figure 5A). As expected, immunofluorescence analysis of TGF-β signalling showed a significant decrease in p-SMAD2/3 expression after galunisertib exposure that was mostly unchanged after sorafenib treatment (Figure 5B). P-AKT and p-ERK staining were both decreased in 60% of the samples when treated with galunisertib (6 out of 10 samples displayed >25% decrease in protein expression) and in 90% (9/10) when treated by sorafenib alone. Interestingly, p-SMAD2/3, p-AKT, and p-ERK staining were strongly inhibited in all samples when treated by a combination of galunisertib and sorafenib. Overall, short exposure of patients’ samples to galunisertib, sorafenib, or a combination of both treatments reduced proliferation and increased apoptosis. All 3 treatments affected noncanonical TGF-β signalling. Potentiation of sorafenib effects on PI3K/AKT and MAPK signalling pathways by galunisertib suggests potential antiproliferative synergy in long-term exposure. Overall analysis of patient samples demonstrated a response on both proliferation and apoptosis for galunisertib and/or galunisertib/sorafenib combination in 6 out of 13 patients: 02, 03, 05, 07, 09, and 11. In these patients, drugs induced significant proliferation decrease (>10%) and apoptosis induction (>50%). All these patients were characterized for well or moderately differentiated HCC. In contrast, the poorly differentiated HCC sample #12 treated with galunisertib did not respond to the drug. The 2 HCC displaying high AFP levels by IHC were sensitive to the *ex vivo* treatment by galunisertib and its combination with sorafenib. No clear correlation of tumor response with sex, age, etiology, and number of satellite nodules was found in this panel of 13 HCCs.

## DISCUSSION

In the present study, we showed that galunisertib displayed potent inhibition of canonical and noncanonical pathways in a variety of *in vitro* cell-lines models likely representing different types of HCC. Galunisertib displayed potent inhibition of the canonical TGF-β pathway as visualized through inhibition of p-Smad2 expression in all models. It also displayed various degrees of inhibition towards noncanonical TGF-β pathways such as MAPK or PI3K/AKT/mTOR. We also observed that galunisertib inhibition on noncanonical TGF-β signaling could be TGF-β independent. Interestingly, the SK-Sora cell lines displayed enhanced canonical and noncanonical TGF-β pathway inhibition suggesting that sorafenib-tolerant cells remain sensitive to TGF-β inhibition. Despite limited antiproliferative activity *in vitro*, we confirmed results from Dituri and colleagues [23] that galunisertib had potent anti-invasive activity and showed that in *ex vivo* models elaborated directly from patients, TGF-β inhibition can display antiproliferative activity. Moreover, in those fresh tumor explants, galunisertib and sorafenib combination displayed enhanced canonical and noncanonical TGF-β pathway inhibition.

Many authors have attempted to refine the molecular classification of HCC and decipher the role of TGF-β in liver carcinogenesis [26, 27]. These studies have highlighted interesting features of HCC tumors that may be relevant to liver carcinogenesis and treatments such as CTNNB1 mutations, Wnt and TGF-β pathway alterations, AFP expression, or virus infection [15, 16, 26-28]. One of the most relevant works for our study came from Coulouarn et al. for its characterization of a HCC TGF-β signature which subdivides HCC into positive and negative TGF-β signature subgroups [15]. Interestingly, within the positive TGF-β signature subgroups, they identified 2 signatures, an “early” and a “late” TGF-β signature that effectively discriminate patients’ populations in 2 very different prognostic groups. Compared to the “early” TGF-β, the “late” TGF-β signature population was significantly associated with shorter survival, increased recurrence, HBV infection, vascular invasion, and a positive c-MET-regulated gene expression signature. In contrast, tumor size, Edmonson grade, or AFP levels were not discriminatory between the 2 populations. In terms of survival and recurrence, the negative TGF-β signature subgroup, which represents approximately 50% of HCC, displayed intermediate risk compared to “early” and “late” subgroups [15]. Work from Hoshida and colleagues [26] showed that the “late” TGF-β signature as defined by Coulouarn and colleagues was strongly associated with one of their 3 HCC subclasses, the S1 subclass characterized by activation of the TGF-β and Wnt pathways whereas their S2 subclass, which is still associated with poor prognostic HCC, was characterized by Myc and AKT activation and positive EpCAM gene signatures as well as elevated serum AFP levels [26]. Authors extended the early and late TGF-β signatures to *in vitro* cell-lines models showing that these models, usually thought to represent late-stage HCC, may in fact reflect various clinical and molecular HCC subclasses and can recapitulate the 2 TGF-β signature subgroups observed in patients [15, 24].

Besides the cell lines that pertain to early TGF-β signature (HepG2, Hep3B, and HuH7), we were most interested by those that were archetypal of the “late TGF-β signature”, *i.e*., SK-HEP1, SK-Suni, and SK-Sora. Those cell lines were invasive, insensitive to the cytostatic effects of TGF-β, but responsive to TGF-β activation and could mimic certain late stages HCC in the clinic, particularly those previously treated with sorafenib. These cells harbored a mesenchymal phenotype expressing Vimentin, Slug, and increased c-MET levels. Our study confirmed previous work on TGF-β inhibitors, including galunisertib, showing that these inhibitors displayed limited antiproliferative effects [23]. In the “early” TGF-β signature models, galunisertib displayed a dose-dependent inhibition of TGF-β dependent cytostasis, whereas in the “late” TGF-β signature models neither TGF-β nor galunisertib displayed anti-proliferative effects. Of note, high galunisertib concentrations (100 μM) seemed to display nonspecific proliferation inhibition affecting most models. In contrast, galunisertib potently inhibited invasion of the mesenchymal cells confirming the results of Dituri and colleagues on HLE and HLF cell lines that both belong to the “late” TGF-β signature subgroup [15, 23].

Many studies have demonstrated the role of TGF-β in stimulating EMT transition [5, 27, 29] that mimics our previous report of a patient with advanced HCC treated with VEGFR inhibitor-displaying tumor morphology reflecting EMT transition [5, 29, 30, 31]. Pathological examination on post-treatment specimens revealed the presence of 2 juxtaposed tissue components containing either sarcomatoid-like mesenchymal cells or well- to moderately differentiated HCC cells with a part of sarcomatoid tissue.

We showed that galunisertib inhibited TGF-β canonical pathway in most HCC cells regardless of their epithelial or mesenchymal differentiation or TGF-β pathway proteins expression. Galunisertib exposure had various effects on noncanonical pathways in cells previously stimulated by TGF-β that were enhanced at higher concentrations and longer exposure time. Interestingly, noncanonical TGF-β signalling in cell lines with acquired resistance to sorafenib seemed to be particularly sensitive to galunisertib.

It has long been recognized that the role of TGF-β signaling in carcinogenesis is dependent and executed at both tumor and tumor microenvironment levels. This is especially true in HCC, in which tumors mostly develop on pathologic livers. It is thus difficult to reach definitive conclusion from *in vitro* cell-line experiments because of the importance of strong risk factors related to the HCC environment such as vascular invasion or the underlying liver pathology such as cirrhosis or fibrosis. Besides, other elements of the liver microenvironment have been shown to play a role in HCC development such as activated pericytes or immune cells that expressed many pro-oncogenic growth factors and cytokines. This is the reason why we chose to test galunisertib in a more physiological context, in which fresh tumor slices directly extracted from patients are maintained alive in their own tissue microenvironment. In these tissues, we confirmed that exposure *ex vivo* samples to galunisertib for 48 hours led to significant inhibition of canonical (p-Smad2) and noncanonical (p-AKT, p-ERK) TGF-β pathway signaling. Moreover, galunisertib induced decreased proliferation markers and increased apoptosis. Sorafenib treatment alone also decreased proliferation and induced apoptosis. Combination of sorafenib with galunisertib increased the effects on cell-signalling inhibition and consequently the antiproliferative and pro-apoptotic effects of the treatment. Interestingly, these effects were independent of AFP expression level. All these data may reinforce the basis for clinical use of galunisertib in combination with sorafenib in HCC patients.

Galunisertib is currently under clinical investigation in HCC patients in second line after sorafenib failure or in patients ineligible for sorafenib. Preliminary results from a phase II clinical trial have shown improved clinical outcome and also changes consistent with a reduction of EMT [32, 33]. Extension of the study to galunisertib in combination with sorafenib in first line is currently in progress (NCT01246986).

In conclusion, we showed that TGF-β inhibition using galunisertib is effective *in vitro* on invasive tumor models as well as *ex vivo* alone or in combination with sorafenib. Combination with sorafenib exacerbated canonical and noncanonical TGF-β pathway inhibition. These data support the use of TGF-β inhibition in advanced HCC with a positive perspective regarding its potential combination with sorafenib in the clinic.

## MATERIALS AND METHODS

### Cell lines

Hepatocellular carcinoma SK-HEP1, HepG2, Hep3B cell lines were obtained from the ATCC (Rockville, MD). JHH6 and HuH7 cell lines were purchased from JCRB cell bank, Japan. SK-Suni and SK-Sora are variants of SK-HEP1 exposed to stepwise increasing concentrations of sunitinib or sorafenib (0.1-10 μM) for more than 6 months. Cells were grown as monolayers in RPMI medium supplemented with 10% fetal calf serum (Invitrogen, Cergy-Pontoise, France), 2 mM glutamine, 100 units/mL penicillin and 100 μg/mL streptomycin at 37°C in a humidified 5% CO_2_ atmosphere and regularly checked for the absence of Mycoplasma.

### Cell cytotoxicity assay

Cell survival was determined using the MTT assay (3-[4,5-dimethylthiazol-2-yl]-2,5-diphenyltetrazolium bromide; Sigma, Saint-Quentin Fallavier, France). The conversion of yellow water-soluble tetrazolium MTT into purple insoluble formazan is catalyzed by mitochondrial dehydrogenases and used to estimate the number of viable cells. In brief, cells were seeded in 96-well tissue culture plates at a density of 2 × 10^3^ cells/well. After drug exposure, cells were incubated with 0.4 mg/mL MTT for 4 hours at 37°C. After incubation, the supernatant was discarded, insoluble formazan precipitates were dissolved in 0.1 mL of DMSO, and the absorbance was measured at 560 nm by use of a microplate reader (Thermo, France). Wells with untreated cells or with drug-containing medium without cells were used as positive and negative controls respectively. For proliferation assay, MTT assay was done daily to determine the number of viable cells in untreated control and galunisertib-treated group. Galunisertib was supplied by Eli Lilly. Human TGF-β1 was obtained from R&D Systems.

### Real-time quantitative RT-PCR

The theoretical and practical aspects of real-time quantitative RT-PCR using the ABI Prism 7900 Sequence Detection System (Perkin-Elmer Applied Biosystems, Foster City, CA, USA) have been described in detail elsewhere. Results were expressed as n-fold differences in target gene expression relative to the TBP gene (an endogenous RNA control) and relative to a calibrator (1X sample), consisting of the cell-line sample from our tested series that contained the smallest amount of target gene mRNA. Experiments were performed in duplicate.

### Western blot analysis

Cells were lysed in buffer containing 50 mM HEPES (pH 7.6), 150 mM NaCl, 1% Triton X-100, 2 mM sodium vanadate, 100 mM NaF, and 0.4 mg/mL phenylmethylsulfonyl fluoride. Equal amounts of protein (30μg/lane) were subjected to SDS-PAGE and transferred to nitrocellulose membranes. Membranes were blocked with 5% milk in 0.05% Tween 20/phosphate-buffered saline and then incubated with the primary antibody overnight. Membranes were then washed and incubated with the secondary antibody conjugated to horseradish peroxidase. Bands were visualized by using the enhanced chemiluminescence Western blotting detection system. Densitometric analysis was performed under conditions that yielded a linear response.

### Modified OptiCell invasion assay

Dermal fibroblasts were embedded in a collagen gel at a density of 10^5^ fibroblasts/ml and then injected vertically on Nunc OptiCell system (Thermo fisher scientific, Waltham, MA) until its polymerization. SK-HEP1, SK-Suni, and SK-Sora cancer cells were then added on top of the gel at a density of 10^5^ cells/ml. The whole system was incubated for 1 week at 37°C in humidified atmosphere with 5% CO_2_, and cancer cell migration was analyzed daily using epifluorescent microscopy.

### *Ex Vivo* tissue profiling (TIPCAN^®^)

The effects of galunisertib were tested on freshly resected tumors from HCC patients which can be cultured alive in specific conditions of culture medium and atmosphere, depending on available tumor resection from the surgical department. After pathological evaluation by the hospital pathologist, the tumor samples were extemporaneously sliced using Tissue Slicer® instrument into 300μm-thick slices and cultured “alive” at 37°C into the William’s E medium, complemented with in-house proprietary dedicated components including foetal calf serum, glucose, gentamicin and HEPES, under normoxic conditions. The samples were prepared using tissue-slicer technology and treated for 24 to 72 hours with 1 and 10 μM galunisertib or 5 μM sorafenib. After 24 to 72 hours treatment, the explanted HCC was paraffin embedded and assessed for expression of selected markers. The tests comprised assessment of cancer cell proliferation (MIB1/Ki67), death (active caspase- 3), and several changes in cell signalling (phospho-kinases). Tissue quality was assessed by a pathologist. If tissue integrity was not maintained over time (>20% necrosis induction), tissues were discarded.

### Immunofluorescence

Tumor slices were fixed in 4% of paraformaldhyde for 15 minutes. Incubation with primary antibodies p-SMAD2/3, p-AKT, p-ERK1/2 (Cell Signaling) was performed at 4°C overnight, followed by incubation with the secondary antibody (anti- Alexa Fluor® 488 Rabbit IgG (H+L) F(ab’)2, Cell Signaling, France) for 1 hour at room temperature in the dark. The nuclei were stained with Dapi 1:20000 (Santa Cruz Biotechnology, Santa Cruz, California, USA). The images were captured and analyzed with a Zeiss observer Z1 microscope. Images quantification was performed using Histolab Software (Microvision, France) subtracting the background.

### Immunohistochemistry

The immunohistochemical (IHC) procedure was performed on paraffin-embedded tumor samples. MIB1/Ki67, caspase-3, c-MET and AFP stainings were performed using automat after the standard procedure used for clinical samples. The images were captured and analyzed with a Zeiss observer Z1 microscope. Images quantification was performed using Histolab Software (Microvision, France) subtracting the background.

## SUPPLEMENTARY FIGURES AND TABLE



## References

[1] World Health Organization (WHO) Cancer. http://www.who.int/mediacentre/factsheets/fs297.

[2] Llovet JM, Ricci S, Mazzaferro V, Hilgard P, Gane E, Blanc JF, de Oliveira AC, Santoro A, Raoul JL, Forner A, Schwartz M, Porta C, Zeuzem S (2008). Sorafenib in advanced hepatocellular carcinoma. N Engl J Med.

[3] Cheng AL, Kang YK, Chen Z, Tsao CJ, Qin S, Kim JS, Luo R, Feng J, Ye S, Yang TS, Xu J, Sun Y, Liang H (2009). Efficacy and safety of sorafenib in patients in the Asia-Pacific region with advanced hepatocellular carcinoma: a phase III randomised, double-blind, placebo-controlled trial. Lancet Oncol.

[4] Yingling JM, Blanchard KL, Sawyer JS (2004). Development of TGF-beta signalling inhibitors for cancer therapy. Nat Rev Drug Discov.

[5] Bierie B, Moses HL (2006). Tumour microenvironment:TGFbeta: the molecular Jekyll andHyde of cancer. Nat Rev Cancer.

[6] Breuhahn K, Longerich T, Schirmacher P (2006). Dysregulation of growth factor beta signalling in hepatocellular carcinoma. Oncogene.

[7] Neuzillet C, de Gramont A, Tijeras-Raballand A, de Mestier L, Cros J, Faivre S, Raymond E (2014). Perspectives of TGF-β inhibition in pancreatic and hepatocellular carcinomas. Oncotarget.

[8] Yakicier MC, Irmak MB, Romano A, Kew M, Ozturk M (1999). Smad2 and Smad4 gene mutations in hepatocellular carcinoma. Oncogene.

[9] Furuta K, Misao S, Takahashi K, Tagaya T, Fukuzawa Y, Ishikawa T, Yoshioka K, Kakumu S (1999). Gene mutation of transforming growth factor beta1 type II receptor in hepatocellular carcinoma. Int J Cancer.

[10] Mamiya T, Yamazaki K, Masugi Y, Mori T, Effendi K, Du W, Hibi T, Tanabe M, Ueda M, Takayama T, Sakamoto M (2010). Reduced transforming growth factor-beta receptor II expression in hepatocellular carcinoma correlates with intrahepatic metastasis. Lab Invest.

[11] Yao L, Li FJ, Tang ZQ, Gao S, Wu QQ (2012). Smad4 expression in hepatocellular carcinoma, differs by hepatitis status. Asian Pac J Cancer Prev.

[12] Torbenson M, Marinopoulos S, Dang DT, Choti M, Ashfaq R, Maitra A, Boitnott J, Wilentz RE (2002). Smad4 overexpression in hepatocellular carcinoma is strongly associated with transforming growth factor beta II receptor immunolabeling. Hum Pathol.

[13] Hiwatashi K, Ueno S, Sakoda M, Kubo F, Tateno T, Kurahara H, Mataki Y, Maemura K, Ishigami S, Shinchi H, Natsugoe S (2009). Strong Smad4 expression correlates with poor prognosis after surgery in patients with hepatocellular carcinoma. Ann Surg Oncol.

[14] Xia H, Ooi LL, Hui KM (2013). MicroRNA-216a/217-induced epithelial-mesenchymal transition targets PTEN and SMAD7 to promote drug resistance and recurrence of liver cancer. Hepatology.

[15] Coulouarn C, Factor VM, Thorgeirsson SS (2008). Transforming growth factor-beta gene expression signature in mouse hepatocytes predicts clinical outcome in human cancer. Hepatology.

[16] Laurent-Puig P, Zucman-Rossi J (2006). Genetics of hepatocellular tumors. Oncogene.

[17] Fransvea E, Mazzocca A, Antonaci S, Giannelli G (2009). Targeting transforming growth factor (TGF)-betaRI inhibits activation of beta1 integrin and blocks vascular invasion in hepatocellular carcinoma. Hepatology.

[18] Mima K, Hayashi H, Kuroki H, Nakagawa S, Okabe H, Chikamoto A, Watanabe M, Beppu T, Baba H (2013). Epithelial-mesenchymal transition expression profiles as a prognostic factor for disease-free survival in hepatocellular carcinoma: Clinical significance of transforming growth factor-beta signaling. Oncol Lett.

[19] Bedossa P, Peltier E, Terris B, Franco D, Poynard T (1995). Transforming growth factor-beta 1 (TGF-beta 1) and TGF-beta 1 receptors in normal, cirrhotic, and neoplastic human livers. Hepatology.

[20] Giannelli G, Mazzocca A, Fransvea E, Lahn M, Antonaci S (2011). Inhibiting TGF-β signaling in hepatocellular carcinoma. Biochim Biophys Acta.

[21] Fransvea E, Angelotti U, Antonaci S, Giannelli G (2008). Blocking transforming growth factor-beta up-regulates E-cadherin and reduces migrations and invasion of hepatocellular carcinoma cells. Hepatology.

[22] Mu Y, Gudey SK, Landstrom M (2012). Non-Smad signaling pathways. Cell Tissue Res.

[23] Dituri F, Mazzocca A, Peidrò FJ, Papappicco P, Fabregat I, De Santis F, Paradiso A, Sabbàgrave; C, Giannelli G (2013). Differential Inhibition of the TGF-β Signaling Pathway in HCC Cells Using the Small Molecule Inhibitor LY2157299 and the D10 Monoclonal Antibody against TGF-β Receptor Type II. PLoS One.

[24] Dzieran J, Fabian J, Feng T, Coulouarn C, Ilkavets I, Kyselova A, Breuhahn K, Dooley S, Meindl-Beinker NM (2013). Comparative analysis of TGF-β/Smad signalling dependent cytostasis in human hepatocellular carcinoma cell lines. PLoS One.

[25] Guorguieva I, Cleverly AL, Stauber A, Sada Pillay N, Rodon JA, Miles CP, Yingling JM, Lahn MM (2014). Defining a therapeutic window for the novel TGF-β inhibitor LY2157299 monohydrate based on a pharmacokinetic/pharmacodynamic model. Br J Clin Phamacol.

[26] Hoshida Y, Nijman SM, Kobayashi M, Chan JA, Brunet JP, Chiang DY, Villanueva A, Newell P, Ikeda K, Hashimoto M, Watanabe G, Gabriel S, Friedman SL (2009). Integrative Transcriptome Analysis Reveals Common Molecular Subclasses of Human Hepatocellular Carcinoma. Cancer Res.

[27] Lachenmayer A, Alsinet C, Savic R, Cabellos L, Toffanin S, Hoshida Y, Villanueva A, Minguez B, Newell P, Tsai HW, Barretina J, Thung S, Ward SC (2012). Wnt-pathway activation in two molecular classes of hepatocellular carcinoma and experimental modulation by sorafenib. Clin Cancer Res.

[28] Guichard C, Amaddeo G, Imbeaud S, Ladeiro Y, Pelletier L, Maad IB, Calderaro J, Bioulac-Sage P, Letexier M, Degos F, Clément B, Balabaud C, Chevet E (2012). Integrated analysis of somatic mutations and focal copy-number changes identifies key genes and pathways in hepatocellular carcinoma. Nat Genet.

[29] Xu J, Lamouille S, Derynck R (2009). TGF-beta-induced epithelial to mesenchymal transition. Cell Res.

[30] Marijon H, Dokmak S, Paradis V, Zappa M, Bieche I, Bouattour M, Raymond E, Faivre S (2011). Epithelial-to-mesenchymal transition and acquired resistance to sunitinib in a patient with hepatocellular carcinoma. J Hepatol.

[31] Fransvea E, Angelotti U, Antonaci S, Giannelli G (2008). Blocking transforming growth factor-beta up-regulates E-cadherin and reduces migration and invasion of hepatocellular carcinoma cells. Hepatology.

[32] Giannelli G, Villa E, Lahn M (2014). Transforming Growth Factor-β as a Therapeutic Target in Hepatocellular Carcinoma. Cancer Res.

[33] Faivre S, Santoro A, Kelley RK, Merle P, Gane E, Douillard JY, Waldschmidt D, Mulcahy MF, Costentin C, Minguez B, Papappicco P, Gueorguieva I (2014). A phase 2 study of a novel transforming growth factor-beta (TGF-β1) receptor I kinase inhibitor, LY2157299 monohydrate (LY), in patients with advanced hepatocellular carcinoma (HCC). J Clin Oncol.

