# Supplementary material for: Effects of TGF-beta signalling inhibition with galunisertib (LY2157299) in hepatocellular carcinoma models and in ex vivo whole tumor tissue samples from patients
Source: Oncotarget. 2015 May 27;6(25):21614–27. doi: 10.18632/oncotarget.4308 (PMC4673290; doi:10.18632/oncotarget.4308)
Supplement: Supplementary file 1 [file oncotarget-06-21614-s001.pdf]

Effects of TGF-beta signalling inhibition with galunisertib (LY2157299) in hepatocellular carcinoma models and in *ex vivo* whole tumor tissue samples from patients

Supplementary Material

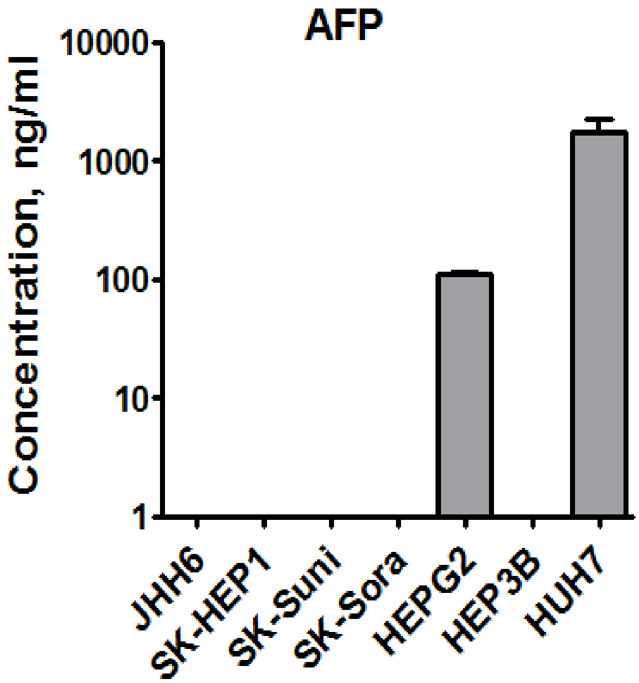

Supplementary Figure 1: Concentration of AFP in conditioned medium of HCC cells

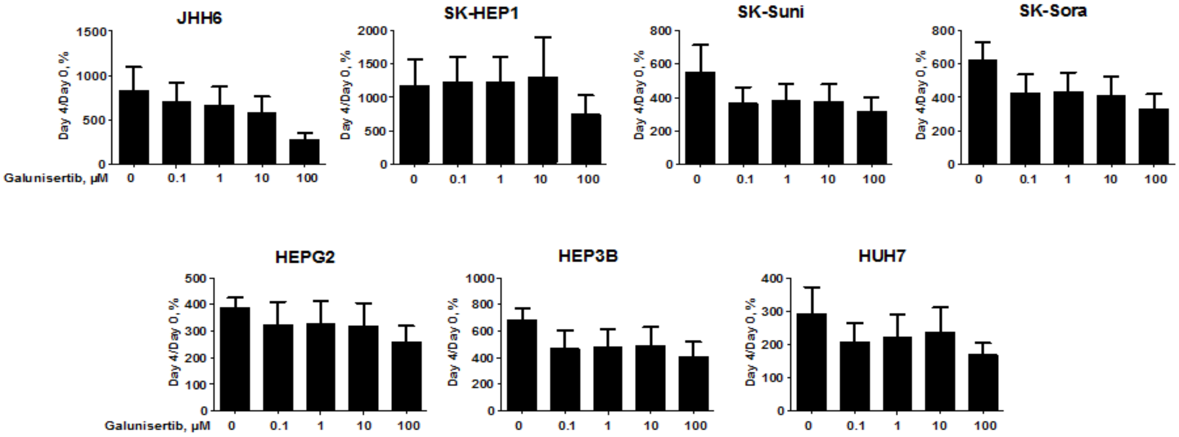

Supplementary Figure 2: Antiproliferative effects of galunisertib without TGF-β on a panel of HCC cell lines.

Patient ID	Proliferation (%)			Apoptosis (%)			pSMAD (%)			pAKT (%)			pERK (%)		
	Galunisertib	Sora	G+Sora	Galunisertib	Sora	G+Sora	Galunisertib	Sora	G+Sora	Galunisertib	Sora	G+Sora	Galunisertib	Sora	G+Sora
**570725**	-98,3	-89,6	-	-53,7	800,2	-	-93,1	0,7	-	-74,9	-84,0	-	-28,2	-73,2	-
**570790**	-97,9	-89,8	-	1850,7	543,7	-	-74,3	-17,8	-	-	-	-	-	-	-
**590914**	-99,4	-78,4	-	3165,7	7625	-	-87,0	-11,0	-	-78,9	-84,6	-	-26,7	-97,3	-
**3570**	54,5	111,9	-	110,3	55,2	-	-	-	-	-	-	-	-	-	-
**3033**	-30,9	-33,1	-55,8	13,4	4,1	46,7	-64,4	-8,6	-89,6	-59,7	-57,7	-81,2	-62,2	-77,9	-86,0
**8590**	9,9	-27,6	-65,6	0,8	145,0	200,9	-39,7	63,0	-87,3	9,3	-39,5	-80,6	-19,4	-59,0	-90,6
**8321**	-30,7	-28,7	-40,8	57,9	130,8	220,2	-75,3	-40,6	-91,8	-8,7	-53,2	-80,7	-38,3	-90,4	-71,4
**5659**	4,3	-48,9	-41,7	42,7	-6,2	-3,8	-78,5	-13,1	-95,9	-22,4	-73,3	-93,6	-16,6	-60,5	-79,6
**5916**	-10,0	-28,1	-60,3	74,0	322,6	187,7	-44,7	-16,2	-83,3	-48,0	-25,5	-75,6	-57,9	-35,5	-86,5
**9951**	-10,4	-13,9	7,6	14,2	-40,0	-37,7	-66,7	-13,6	-91,5	-12,4	-21,2	-67,0	-68,8	-53,0	-63,3
**9161**	-30,9	-34,7	-27,6	69,5	100,8	93,3	-51,1	-4,8	-89,5	-45,8	-50,3	-87,5	-36,0	-63,4	-90,7
**9160**	56,8	-37,5	15,1	-13,3	-29,9	-84,4	-63,0	0,3	-86,5	-36,8	-43,9	-68,8	-21,1	-48,9	-74,5
**9472**	-36,4	-8,3	-19,2	-39,3	11,2	-37,2	-72,3	-13,2	-77,9	-33,5	-51,8	-90,1	-17,8	-16,0	-77,9

Supplementary Table 1. Relative levels of Ki67/Mib1, caspase-3, p-SMAD2/3, p-AKT, and p-ERK1/2 in patient samples treated *ex vivo* with galunisertib (G), sorafenib (Sora), or a combination of both treatments (G+Sora).

Percentages represent the protein expression relative to control after 48 hours of culture.
